# Hydrotherapy for Overweight and Obesity: A Systematic Review of Anthropometric and Lipid Outcomes

**DOI:** 10.7759/cureus.115048

**Published:** 2026-08-23

**Authors:** Mohanakrishnan B, Anvesh Barakoti, Ananya Prasad, Reet Arora, Rithik Narayanan, Arkaprava Das Adhikary

**Affiliations:** 1 Department of Naturopathy and Yoga, Maharishi Aurobindo Subharti College and Hospital of Naturopathy and Yogic Sciences, Swami Vivekanand Subharti University, Meerut, IND

**Keywords:** anthropometric outcomes, aquatic exercise, hydrotherapy, lipid profile, obesity, sauna bathing

## Abstract

Overweight and obesity are chronic metabolic conditions associated with excess adiposity, impaired physical function, dyslipidemia, and increased cardiometabolic risk. Among hydrotherapy-based modalities, structured aquatic exercise may provide a low-impact exercise option by reducing weight-bearing joint loading, providing water resistance, and facilitating exercise participation, whereas passive hydrothermal interventions and hydrogen-rich water involve different therapeutic mechanisms. This systematic review evaluated the effects of hydrotherapy-based and water-based interventions on anthropometric and lipid profile outcomes in individuals with overweight or obesity. A systematic search was conducted in the Cochrane Central Register of Controlled Trials (CENTRAL), Google Scholar, MEDLINE, PubMed, and Scopus as the main sources, with Web of Science and ScienceDirect as additional sources. MEDLINE and PubMed were retained as separately named prespecified search sources despite their substantial overlap. Searches were conducted without time limits during the registered review period from June 23 to July 31, 2026. Published English-language human intervention studies were eligible if they evaluated hydrotherapy, aquatic exercise, aqua aerobics, water-based aerobic training, aquatic high-intensity interval training, balneotherapy, sauna-based therapy, steam bath, dry sauna, immersion-based therapy, or hydrogen-rich water and reported at least one extractable anthropometric or lipid outcome. Ten studies were included in the qualitative synthesis. Active water-based exercise interventions showed the most consistent pattern of improvement in body weight, body mass index, waist-related measures, body fat percentage, and fat mass. In one randomized double-blind placebo-controlled study, hydrogen-rich water was associated with reductions in body mass index, waist-to-hip ratio, total cholesterol, and triglycerides. Passive hydrothermal interventions showed more variable anthropometric and lipid findings. Reported safety findings were generally reassuring, although adverse-event, adherence, and withdrawal reporting was inconsistent. Hydrotherapy-based interventions may have a complementary role in obesity management. Within the available evidence, structured active aquatic exercise demonstrated the most consistent pattern of anthropometric improvement; however, the certainty of evidence across most outcome domains was low to low-to-moderate, warranting cautious interpretation.

## Introduction and background

Overweight and obesity are significant public health problems with substantial clinical, metabolic, functional, and socioeconomic implications [1]. Recent global evidence indicates that more than one billion people were living with obesity in 2022, and population-level analyses across 200 countries and territories show that the increasing global burden of abnormal body weight has been driven predominantly by rising obesity prevalence [1,2]. Hyperadiposity is linked with problems with mobility, lowered cardiorespiratory fitness, musculoskeletal stress, insulin resistance, dyslipidemia, hypertension, chronic inflammatory markers, and increased cardiometabolic risk [3]. The clinical consequences are not limited to weight and include changes in body composition, central fat distribution, impaired physical functioning, decreased quality of life, and increased risk of chronic non-communicable diseases [4]. Body mass index (BMI) continues to be used, but waist circumference, hip circumference, waist-to-hip ratio (WHR), body fat percentage, fat mass, lean mass, visceral fat, and skinfold thickness are other measures that give information about body adiposity distribution and metabolic risk [5]. People with overweight or obesity are likely to have lipid abnormalities. Elevated total cholesterol, triglycerides, low-density lipoprotein (LDL) cholesterol (LDL-C), very-LDL (VLDL) cholesterol (VLDL-C), and reduced high-density lipoprotein (HDL) cholesterol (HDL-C) contribute to atherogenic risk and adverse cardiovascular outcomes [6]. In addition to weight loss, BMI reduction, waist-related measurements, and fat loss, an improvement in the lipid profile is an important goal to be achieved in obesity treatment [7]. Any intervention that positively affects anthropometric and lipid parameters can yield clinically meaningful changes, especially in those who are unable to engage in the same level of physical activity, have limited joint mobility, or experience fatigue or discomfort in their joints [8].

Exercise is an integral part of non-pharmacological treatment for obesity, and lifestyle modification is a key factor in the management of obesity [3]. The standard exercise program offered on the ground can be challenging for some people with excess body weight because of joint loading, musculoskeletal pain, limited balance, low exercise tolerance, or lack of motivation to exercise [9]. Hydrotherapy-based and water-based interventions provide an alternative therapeutic setting. Buoyancy decreases apparent body weight and may reduce weight-bearing stress on the joints; water resistance encourages low-impact muscle activity, and hydrostatic pressure can affect venous return, blood flow, and perceived exertion [10]. Thermal exposure via sauna, steam bath, balneotherapy, or immersion therapies can also impact short-term fluid balance, vascular function, relaxation, sweating, and autonomic tone. These physiological characteristics could be beneficial in people with overweight or obesity and make hydrotherapy a possible complementary therapy [11]. Exercise interventions based on water are aqua aerobics, aquatic aerobic training, aquatic high-intensity interval training (HIIT), and continuous or intermittent water exercise. These programs include aerobic challenge, water resistance, and decreased mechanical loading [8]. These attributes can contribute to enhanced body composition, waist measurements, cardiorespiratory fitness, and certain lipid markers [12]. Passive hydrotherapeutics, such as balneotherapy, steam baths, sauna baths, dry saunas, and thermal water exposure, are very different from active aquatic exercise. Such interventions can have an effect on comfort, relaxation, quality of life, and possibly selected metabolic markers; effects on sustained fat loss and lipid modification can be variable [5]. Hydrogen-rich water is another emerging intervention category with potential antioxidant and metabolic properties in people at risk of developing cardiometabolic diseases as part of obesity [9].

The evidence of hydrotherapy for overweight and obesity is still highly variable. The studies available vary in population features, age range, definition of obesity, intervention format, frequency of intervention, length of intervention, type of comparison, and outcome measures. Some interventions include a supervised structured exercise program, while others are passive thermal exposure or the consumption of hydrogen-rich water [13]. Follow-up durations may include immediate post-intervention assessment or several months. Though outcomes of the anthropometric parameters are frequently reported, outcomes of the lipid profile are less consistently assessed [10]. Data on safety and tolerability, adherence, completion, and withdrawal are also inconsistently reported. These variations make it hard to interpret and to compare interventions directly [6]. Given the increasing global burden of obesity and the need for feasible adjunctive approaches for individuals who may have difficulty performing conventional land-based exercise, a focused synthesis of the available hydrotherapy evidence is clinically relevant. More synthesis is required to establish a direction and consistency of effects within the hydrotherapy-based modalities in people with overweight or obesity. The assessment of anthropometric and lipid results should be done separately because a lowering of BMI or measurement of waist might not always be associated with an improvement in lipids. Distinguishing active water-based exercise from passive hydrothermal intervention may also help the clinical interpretation. A systematic review can provide a structure to the evidence available, highlight categories of interventions that show more robust signals of outcomes, assess the methodological quality of the evidence, and delineate areas in which further evidence is needed and should be conducted in a more standardized manner.

Objectives of the review

This systematic review aimed to evaluate the effects of hydrotherapy-based and water-based interventions on anthropometric outcomes in individuals with overweight or obesity. The review also aimed to assess changes in lipid profile parameters and summarize reported findings on adverse events, tolerability, adherence, completion, and withdrawals.

## Review

Methodology

Study Design and Registration

This systematic review aimed to assess the impact of different types of hydrotherapy and water-based interventions on anthropometric and lipid profile outcomes in people with overweight or obesity. This review is registered in PROSPERO (International Prospective Register of Systematic Reviews) under the title “Hydrotherapy for overweight and obesity: a systematic review of anthropometric and lipid outcomes,” with registration number CRD420261423192. A systematic approach was used to review, and the Preferred Reporting Items for Systematic Reviews and Meta-Analyses (PRISMA) framework was followed to report the review [13]. There was variability in study design, type of intervention, comparator group, duration of the intervention, characteristics of the participants, and reporting of outcomes, which warranted a qualitative synthesis of the results of the studies.

Eligibility Criteria

Eligibility was determined according to the registered population, intervention, comparator, study design, context, outcome, publication status, and language criteria. The complete inclusion and exclusion criteria are presented in Table 1. Studies were required to report at least one extractable anthropometric or lipid profile outcome.

**Table 1:** Inclusion and exclusion criteria

Criterion	Inclusion criteria	Exclusion criteria
Population	Human participants of any age with overweight, obesity, morbid obesity, excess body weight, increased body mass index, central adiposity, or obesity-related anthropometric characteristics. Participants with metabolic syndrome were eligible only when overweight, obesity, central adiposity, excess body weight, or obesity-related anthropometric outcomes were clearly reported.	Populations unrelated to overweight, obesity, morbid obesity, adiposity, central adiposity, excess body weight, or obesity-related anthropometric outcomes.
Intervention	Hydrotherapy, aquatic exercise, aqua aerobics, water-based aerobic training, aquatic high-intensity interval training, balneotherapy, sauna therapy, dry sauna therapy, steam bath therapy, thermal water therapy, immersion-based therapy, or hydrogen-rich water intervention.	Interventions that were not hydrotherapy-based, water-based, immersion-based, thermal-water-based, sauna-based, steam-based, or hydrogen-rich-water-based.
Comparator	Usual care or another study-defined comparator, where applicable. Eligible uncontrolled pre-post studies were not required to include a separate comparator.	No exclusion was made solely because a comparator was absent when the study was an eligible uncontrolled pre-post intervention study.
Study design	Randomized controlled trials, non-randomized comparative intervention studies, controlled clinical studies, quasi-experimental studies, and uncontrolled pre-post intervention studies.	Reviews, systematic reviews, meta-analyses, editorials, commentaries, conference abstracts, case reports, animal studies, and in vitro studies.
Context	Studies conducted in any geographical location and in healthcare, rehabilitation, community, wellness, or research settings.	No study was excluded solely on the basis of geographical location or eligible setting.
Primary outcomes	Extractable changes in body weight, body mass index, waist circumference, hip circumference, waist-to-hip ratio, body fat percentage, fat mass, lean body mass, visceral fat measures, skinfold thickness, or another clearly reported obesity-related anthropometric measure.	Studies that did not report at least one extractable anthropometric or eligible lipid profile outcome.
Secondary outcomes	Extractable changes in total cholesterol, triglycerides, low-density lipoprotein cholesterol, or high-density lipoprotein cholesterol. Adverse events, tolerability, adherence, intervention completion, and withdrawal rates were extracted when reported.	Studies without extractable anthropometric or lipid profile outcome data.
Publication status	Full-text published studies.	Unpublished studies, conference abstracts, and publications without sufficient extractable outcome data.
Language	Studies published in English.	Studies published in languages other than English.
Publication date	No publication-date restriction.	No study was excluded solely because of its publication year.

Information Sources and Search Strategy

The search strategy combined terms relating to overweight or obesity, hydrotherapy-based or water-based interventions, and anthropometric or lipid profile outcomes. The core Boolean search formula was ("overweight" OR "obesity" OR "obese") AND ("hydrotherapy" OR "aquatic exercise" OR "aqua aerobics" OR "water-based exercise" OR "aquatic HIIT" OR "balneotherapy" OR "sauna" OR "steam bath" OR "immersion therapy" OR "hydrogen-rich water") AND ("body weight" OR "BMI" OR "waist circumference" OR "body fat" OR "fat mass" OR "lipid profile" OR "cholesterol" OR "triglycerides" OR "LDL-C" OR "HDL-C"). Synonymous terms within each concept were combined using OR, while the three principal concepts were combined using AND. The search scope was database-specific, and the Boolean strategy was applied using the searchable bibliographic and indexing fields supported by each information source, with syntax adapted to the requirements of the respective platform. No single universal field designation, such as Topic Search (TS), was applied across all databases. The search was not restricted to article titles alone. The search sources, principal search concepts, restrictions, and supplementary identification methods are presented in Table 2.

**Table 2:** Search strategy used to identify eligible studies CENTRAL: Cochrane Central Register of Controlled Trials

Information source	Status in the registered protocol	Principal search concepts	Language restriction	Date restriction	Additional details
CENTRAL	Main source	Overweight or obesity AND hydrotherapy or water-based intervention AND anthropometric or lipid profile outcome	English	No restriction	Published studies only
Google Scholar	Main source	Overweight or obesity AND hydrotherapy or water-based intervention AND anthropometric or lipid profile outcome	English	No restriction	Published studies only
MEDLINE	Main source	Overweight or obesity AND hydrotherapy or water-based intervention AND anthropometric or lipid profile outcome	English	No restriction	Published studies only
PubMed	Main source	Overweight or obesity AND hydrotherapy or water-based intervention AND anthropometric or lipid profile outcome	English	No restriction	Published studies only
Scopus	Main source	Overweight or obesity AND hydrotherapy or water-based intervention AND anthropometric or lipid profile outcome	English	No restriction	Published studies only
Web of Science	Additional source	Overweight or obesity AND hydrotherapy or water-based intervention AND anthropometric or lipid profile outcome	English	No restriction	Published studies only
ScienceDirect	Additional source	Overweight or obesity AND hydrotherapy or water-based intervention AND anthropometric or lipid profile outcome	English	No restriction	Published studies only
Reference-list screening	Supplementary method	Backward citation searching of eligible studies	Not applicable	Not applicable	Used to identify additional eligible published studies

Study Selection

All identified records were checked for duplication. Following duplicate removal, titles and abstracts were considered for eligibility based on the criteria. Title-and-abstract screening and full-text assessment were conducted independently by at least two reviewers. Full-text articles were then screened for relevance, considering the following factors: population, intervention, study design, language, publication type, and the presence of an outcome. Only studies that reported at least one anthropometric or lipid profile outcome that could be extracted were included. Exclusion at the full-text stage was recorded and was classified as ineligible population or intervention, insufficient extractable outcome data, or non-English publication. Disagreements between reviewers were resolved through discussion and consensus.

Data Extraction

A structured data extraction form was used to collect relevant information from the included studies. Data extraction was performed independently by at least two reviewers, and any disagreements were resolved through discussion and consensus. Extracted information included the author, year of publication, study design, sample size, participant characteristics, intervention type, comparator condition, intervention duration, follow-up period, anthropometric outcomes, lipid profile outcomes, adverse events, tolerability, adherence, withdrawals, and key findings. Anthropometric outcomes included body weight, BMI, waist circumference, hip circumference, WHR, body fat percentage, fat mass, lean mass, visceral fat, and other body composition measures. Lipid outcomes included total cholesterol, triglycerides, LDL-C, HDL-C, and VLDL-C.

Outcomes

The main outcomes measured were changes in anthropometric indicators of overweight and obesity as well as body weight, BMI, waist circumference, hip circumference, WHR, body fat percentage, fat mass, lean mass, visceral fat, and other reported body composition indices. Secondary outcomes included changes in lipid profile parameters (total cholesterol, triglycerides, LDL-C, HDL-C, and VLDL-C). Other outcomes measured were adverse events, tolerability, adherence, intervention completion, and withdrawal rates.

Risk-of-Bias Assessment

Risk of bias was determined based on the study design. Randomized controlled and randomized comparative studies were assessed using the Cochrane Risk of Bias 2 tool [14]. Risk Of Bias In Non-randomized Studies-of Interventions (ROBINS-I) was used to assess non-randomized comparative intervention studies [15], and Joanna Briggs Institute critical appraisal criteria were used for pre-post intervention studies [16]. The assessment comprised a randomization procedure, comparability of the baseline data, confounding, missing outcome data, outcome measurement, and selective reporting. Each study was assigned an overall judgment of low, low to moderate, moderate, or moderate to high risk of bias.

Data Synthesis

Findings were summarized using a narrative approach. Studies were grouped according to intervention type and outcome domain. Active water-based interventions, including aqua aerobics, aquatic HIIT, water-based aerobic training, and continuous or intermittent water exercise, were summarized separately from passive hydrothermal interventions, including balneotherapy, steam bath, sauna bath, and dry sauna. Hydrogen-rich water was considered as a separate intervention category. Anthropometric and lipid profile outcomes were presented independently. A meta-analysis, meta-regression, or pooled effect estimate was not performed because the included studies were clinically and methodologically heterogeneous with respect to participant characteristics and baseline metabolic status, intervention modality, treatment frequency and duration, comparator conditions, concomitant dietary or pharmacological management, outcome definitions, measurement methods, and reporting units. Statistical pooling was therefore considered inappropriate, and no forest plot was generated. Accordingly, studies with clinically distinct interventions or populations were not treated as directly comparable, and findings were interpreted within individual intervention categories and study contexts. Where possible, extractable numerical data were organized into structured tables so that results reported in comparable formats (e.g., same units or similar outcome measures) could be clearly displayed for descriptive comparison across studies. These numerical data were retained as descriptive study-level findings and were not interpreted as pooled treatment effects.

Certainty of Evidence

For the major outcome domains, the certainty of evidence was determined with the GRADE approach [17]. Evaluation of evidence was done for the following: body weight, BMI, waist-related outcomes, body fat or fat mass, total cholesterol, LDL-C, HDL-C, triglycerides, adverse events, tolerability, adherence, and withdrawals. Risk of bias, inconsistency, indirectness, imprecision, and possible publication bias were taken into account for certainty ratings. The certainty of the evidence was assessed as high, moderate, low, and very low.

Results

Search Result

**Figure 1:**
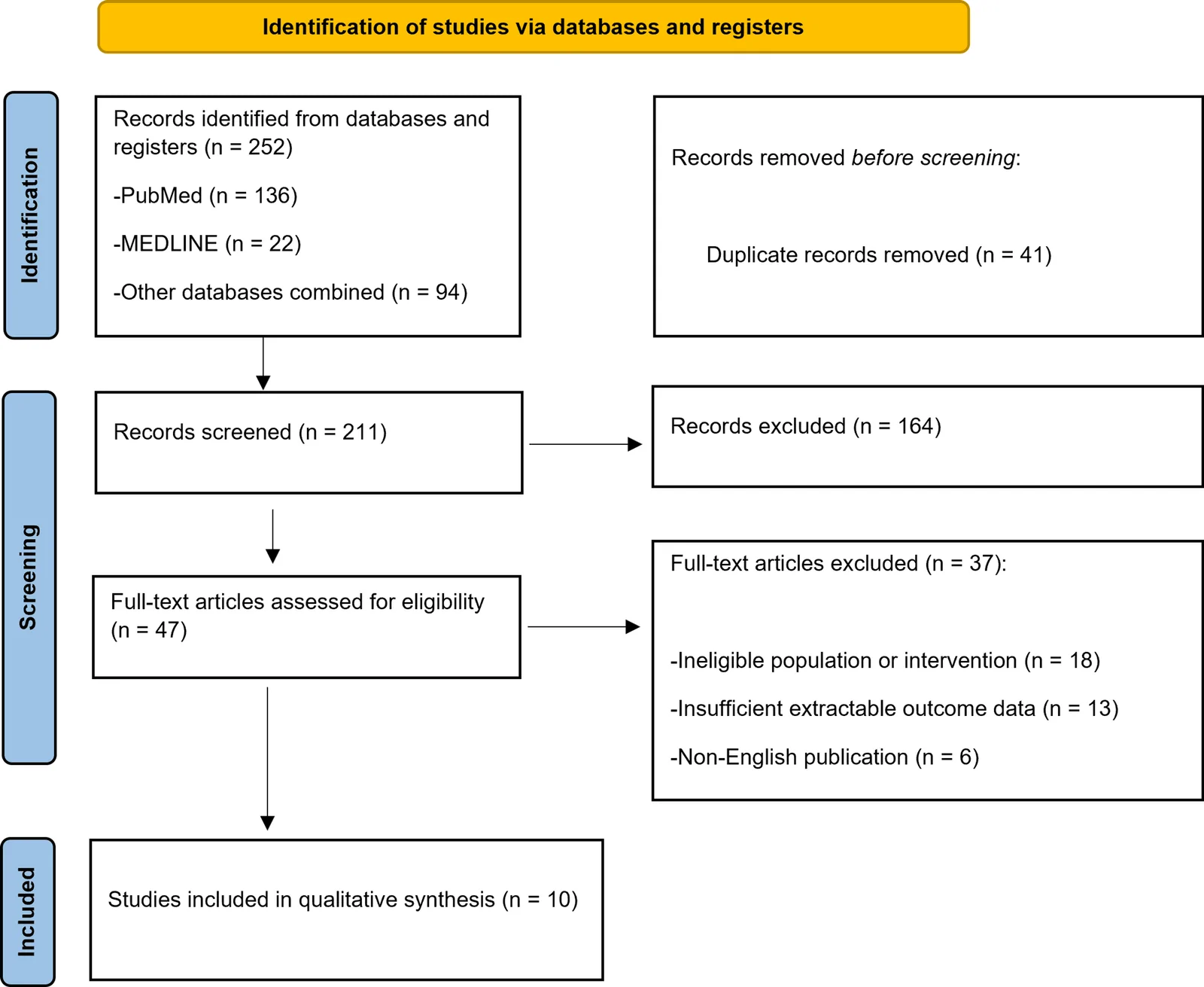
Preferred Reporting Items for Systematic Reviews and Meta-Analyses (PRISMA) flow diagram of study selection

A search of the database resulted in 252 records being found. Of these, 136 records were identified from PubMed, 22 from MEDLINE, and 94 collectively from CENTRAL, Google Scholar, Scopus, Web of Science, and ScienceDirect. Because records from these five sources were documented collectively during the original search, they are presented as a combined total rather than as retrospectively reconstructed database-specific counts. Titles and abstracts were screened for duplicates, and then, full-text eligibility screening was conducted. Records were not included if the study population, intervention, outcome reporting, language, or publication type did not meet the eligibility criteria. The most common causes of full-text exclusions were ineligible intervention/population, lack of extractable anthropometric data, lack of lipid data, and non-English language. Ten studies were selected for qualitative synthesis. Figure 1 reports the identification, screening, eligibility process, reasons for exclusion, and final inclusion of the studies examining the effectiveness of hydrotherapy-based interventions for overweight and obesity.

Study Characteristics

The included studies were clinically diverse among women, obese women, adolescents, the morbidly obese, and participants with metabolic syndrome in adults. Active aquatic exercise programs, aqua aerobics, aquatic HIIT, continuous and intermittent water exercise, balneotherapy, steam bath, sauna bath, dry sauna, and hydrogen-rich water were the different types of interventions. Comparator conditions were usual activity, placebo, baseline status, land-based exercise, or other thermal exposure. Follow-up varied from immediate post-intervention assessment to 24 weeks. Safety reporting was inconsistent, and anthropometric outcomes were more likely to be reported than lipid outcomes. Table 3 shows the characteristics, intervention details, comparator groups, assessed outcomes, and key findings of the included studies.

**Table 3:** Characteristics and key outcomes of included studies BMI: body mass index; LDL-C: low-density lipoprotein cholesterol; HDL-C: high-density lipoprotein cholesterol; VLDL-C: very-low-density lipoprotein cholesterol; HbA1c: glycated hemoglobin; WHR: waist-to-hip ratio; HIIT: high-intensity interval training

Study	Population	Intervention	Comparator	Main outcomes assessed	Key outcomes
Koçak et al. [18]	Women with morbid obesity	Balneotherapy, 15 sessions over three weeks	Pre-intervention baseline	BMI, WHR, body fat percentage, lipid profile, glucose, adipokines, sleep quality, quality of life	Body composition indices showed no significant improvement. Sleep quality and quality-of-life scores improved. LDL-C decreased in the dyslipidemic subgroup, with favorable changes in adipokine profile.
Sobczak et al. [19]	Perimenopausal women with elevated BMI	Aqua aerobics for six months	Control group	Body weight, BMI, fat mass, visceral fat, WHR, lipid profile, blood pressure	Aqua aerobics significantly reduced WHR and LDL-C. Body weight and BMI remained largely unchanged. Diastolic blood pressure improved in the intervention group.
LeBaron et al. [20]	Participants with metabolic syndrome	High-concentration hydrogen-rich water for 24 weeks	Placebo water	BMI, WHR, lipid profile, glucose metabolism, and inflammatory biomarkers	Hydrogen-rich water reduced BMI, WHR, total cholesterol, VLDL-C, triglycerides, glucose, HbA1c, and inflammatory markers. LDL-C showed a modest reduction.
Kantyka et al. [21]	Sedentary middle-aged overweight/obese women	Aqua aerobics, 45 minutes, three times weekly, 14 weeks	Non-exercising control group	Body weight, body composition, total body water, fat-free mass, skeletal muscle mass, lipid profile	Aqua aerobics reduced body weight and body fat percentage and improved skeletal muscle-related measures. Lipid profile changes were small and not statistically significant.
Ben Cheikh et al. [22]	Young, overweight, and obese women	Water-based aerobic training, three times weekly for 10 weeks	Usual activity	Body weight, BMI, body fat percentage, glycemia, total cholesterol, HDL-C, LDL-C, triglycerides	Water-based aerobic training reduced body weight, BMI, body fat percentage, glycemia, LDL-C, and triglycerides, with improvement in HDL-C.
Liao et al. [23]	Obese adolescents	Aquatic high-intensity interval training	Land-based high-intensity interval training	Body mass, BMI, fat percentage, lean mass, waist circumference, hip circumference, WHR, lipid profile	Aquatic HIIT reduced body mass, BMI, fat percentage, waist circumference, hip circumference, WHR, and LDL-C, with increased lean mass.
Penaforte et al. [24]	Obese sedentary women	Continuous water exercise	Intermittent water exercise	Body weight, BMI, body circumferences, fat mass, fat-free mass, glycemia, lipid profile, quality of life	Continuous water exercise reduced weight, BMI, fat mass, arm circumference, and hip circumference. Intermittent water exercise mainly reduced fat mass and arm circumference.
Vasanth Kumar et al. [25]	Obese adults	Steam bath for 10 days	Sauna bath for 10 days	BMI, triglycerides, waist circumference, hip circumference, WHR	Both steam bath and sauna bath reduced BMI, triglycerides, waist circumference, and hip circumference. No serious adverse effects were reported.
Choi et al. [26]	Obese adults	Repeated dry sauna exposure	Pre-intervention baseline	BMI, waist circumference, body composition, blood pressure, heart rate, quality of life, adverse events	Dry sauna improved quality-of-life scores. BMI, waist circumference, body composition, blood pressure, and lipid-related measures showed no meaningful change. No adverse events were reported.
Tripathi et al. [27]	Adults with obesity	Single steam bath session	Single sauna bath session	Body weight, BMI, fat-free mass, total body water, basal metabolic rate, heart-rate variability	Sauna bath produced greater immediate reduction in body weight and BMI. Steam bath produced smaller short-term changes in BMI and body water-related indices.

Risk-of-Bias Assessment

Study design and methodological rigor were found to vary in terms of risk of bias. Randomized and placebo-controlled trials were generally more internally valid, particularly when allocation, blinding, and objective measurement of the outcome were clearly described. Studies that used baseline comparison alone were more likely to be confounded, temporally influenced, subject to regression to the mean, and co-influenced. Outcome measurement concerns included objective measures (BMI, waist circumference, body composition, and serum lipids), with adherence and adverse-event documentation less consistent. The range of risk of bias was from low (in the strongest randomized trial) to moderate to high (in uncontrolled pre-post studies). Table 4 shows the methodological quality and risk-of-bias judgments for the included studies, including the assessment tool applied, key sources of bias, and overall risk classification.

**Table 4:** Risk-of-bias assessment of included studies JBI: Joanna Briggs Institute; RoB 2: Risk of Bias 2; ROBINS-I: Risk Of Bias In Non-randomized Studies-of Interventions

Study	Study design	Assessment tool	Main methodological concerns	Overall judgment
Koçak et al. [18]	Uncontrolled pre-post study	JBI checklist	Absence of control group, confounding, non-randomized design	Moderate to high
Kantyka et al. [21]	Randomized controlled study	RoB 2	Small sample size, unclear allocation concealment, limited blinding	Moderate
Vasanth Kumar et al. [25]	Randomized comparative study	RoB 2	Short intervention duration, unclear blinding, limited allocation details	Moderate
Tripathi et al. [27]	Randomized comparative study	RoB 2	Single-session design, immediate outcome assessment, limited blinding	Moderate
Sobczak et al. [19]	Randomized controlled trial	RoB 2	Small sample size, limited participant blinding	Low to moderate
LeBaron et al. [20]	Randomized double-blind placebo-controlled trial	RoB 2	Minor concerns only	Low
Ben Cheikh et al. [22]	Randomized controlled trial	RoB 2	Small sample size, usual-activity comparator, limited blinding	Low to moderate
Liao et al. [23]	Comparative intervention study	ROBINS-I	Small sample size, behavioral confounding, attrition before completion	Moderate
Penaforte et al. [24]	Comparative water exercise study	ROBINS-I	Dropouts, small sample size, limited dietary control	Moderate
Choi et al. [26]	Uncontrolled pre-post study	JBI checklist	No control group, short duration, non-randomized design	Moderate to high

Effects on Anthropometric Outcomes

**Figure 2:**
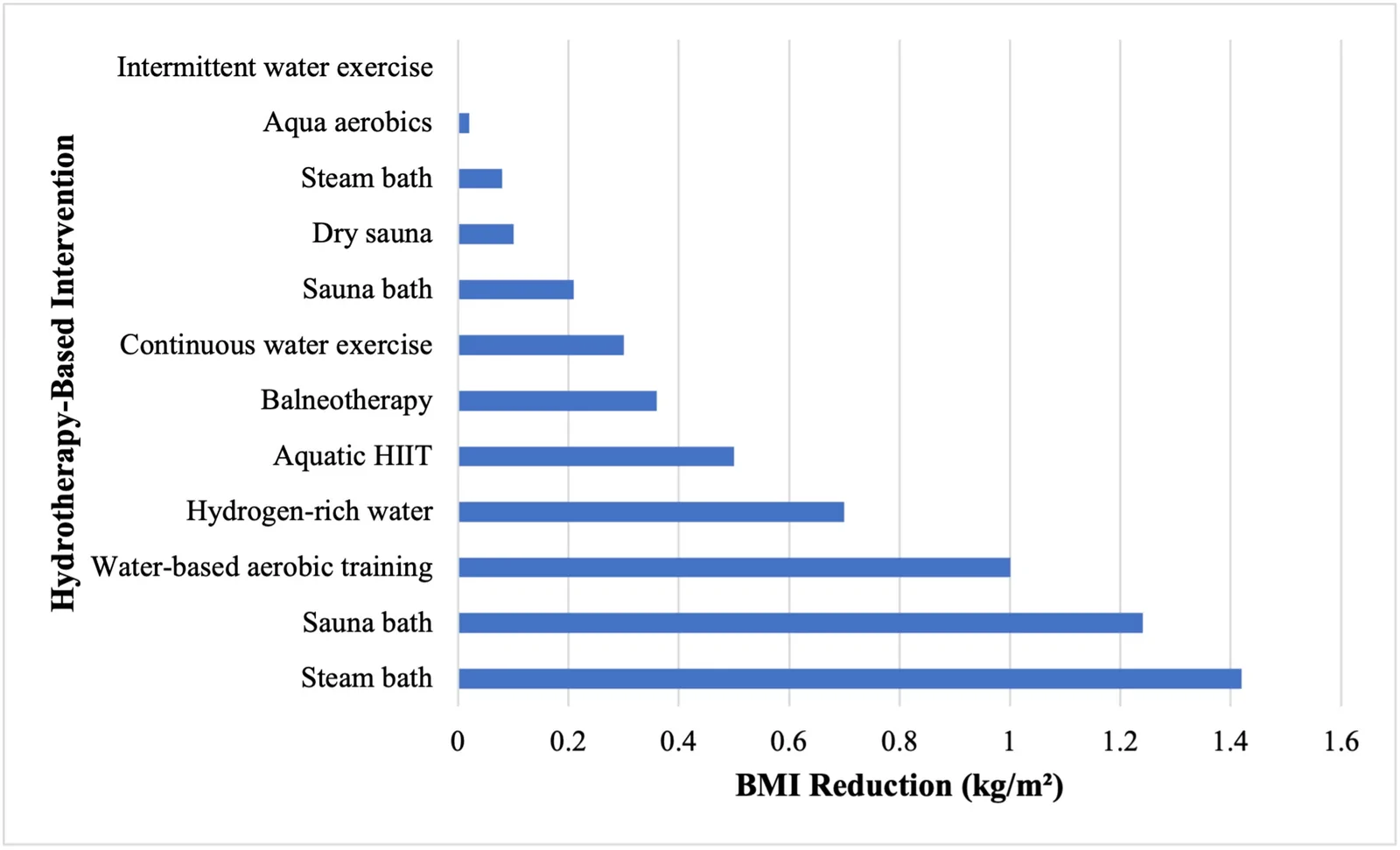
BMI reduction across hydrotherapy-based interventions BMI: body mass index; HIIT: high-intensity interval training Sources: [18-27]

The anthropometric effects varied across intervention types and study durations. Active water-based exercise studies generally reported reductions in body weight, BMI, body fat percentage, fat mass, waist circumference, and WHR; however, the magnitude of these changes varied among studies. Water-based aerobic training and aquatic HIIT were associated with improvements in several body composition parameters within the respective studies. Continuous water exercise showed improvements in a greater number of reported anthropometric measures than intermittent water exercise within the study that directly compared these two approaches. Interventions involving steam and sauna baths also reported short-term changes in BMI and circumference measures. Because the included studies differed in baseline BMI, participant characteristics, intervention duration, study design, and comparator conditions, these findings should not be interpreted as demonstrating the superiority of one hydrotherapy modality over another. Figure 2, therefore, provides a descriptive summary of reported BMI changes across intervention arms rather than a comparative estimate of treatment efficacy.

Effects on Lipid Profile Outcomes

Lipid outcomes were less consistently reported than anthropometric outcomes. Hydrogen-rich water most clearly improved the lipid profile, lowering both total cholesterol and VLDL-C as well as triglycerides. Aerobic training with water did improve HDL-C, LDL-C, and triglycerides. The HIIT performed in water had a significant impact on lowering LDL-C and a smaller impact on lowering total cholesterol, HDL-C, and triglycerides. There was a reduction in LDL-C in the perimenopausal aqua aerobics trial, while other aqua aerobics and water exercise trials had modest and/or not significant reductions in LDL-C. The sauna baths and steam baths were the interventions that primarily decreased triglycerides. Dry sauna did not provide significant improvement in lipids. Table 5 shows the lipid profile changes reported across hydrotherapy-based interventions, including total cholesterol, LDL-C, HDL-C, and triglyceride outcomes.

**Table 5:** Lipid profile outcomes suitable for bar graph preparation LDL-C: low-density lipoprotein cholesterol; HDL-C: high-density lipoprotein cholesterol; NR: not reported

Study/intervention	Total cholesterol change	LDL-C change	HDL-C change	Triglyceride change	Unit	Overall effect
Koçak et al. [18]	NR	-19.90 (127.42 ± 33.76 to 107.52 ± 25.07; p = 0.018) in dyslipidemic subgroup	NR	NR	mg/dL	Selective favorable
Kantyka et al. [21]	-0.23	-0.16	+0.13	-0.13	mmol/L	Small favorable
Vasanth Kumar et al. [25], steam bath	NR	NR	NR	-5.81	mg/dL	Favorable
Vasanth Kumar et al. [25], sauna bath	NR	NR	NR	-5.90	mg/dL	Favorable
Sobczak et al. [19]	-15.01	-11.14	-1.36	+4.72	mg/dL	LDL-C favorable
LeBaron et al. [20]	-18.50	-6.50	-1.30	-47.40	mg/dL	Favorable
Ben Cheikh et al. [22]	-0.22	-0.35	+0.25	-0.29	mmol/L	Favorable
Liao et al. [23]	-0.03	-0.09	+0.01	-0.02	mmol/L	LDL-C favorable
Penaforte et al. [24], steam bath	+1.10	0.00	-1.10	+12.30	mg/dL	Neutral
Penaforte et al. [24], sauna bath	-1.70	-1.10	+0.40	-6.60	mg/dL	Small favorable
Choi et al. [26], dry sauna	NR	NR	NR	NR	NR	Neutral

Adverse Events, Tolerability, Adherence, and Withdrawals

The results of safety and feasibility studies were generally good, with incomplete reporting. Major complications were not found in studies that specifically evaluated adverse events. There were no adverse events reported during exposure to a dry sauna. Interventions with steam and sauna baths were reported without serious complications. Hydrogen-rich water was well accepted, and follow-up of the trials was completed. Aqua aerobics and aquatic HIIT were also suggested to be feasible in supervised environments, though attendance limits and monitoring of adverse events were not always reported. In the comparability study to water exercise and in the study on adolescent HIIT, dropout rates were highlighted before completion, which meant that certainty about adherence was kept limited across all forms of interventions. Table 6 displays the safety, tolerability, adherence, completion, and withdrawal information from the studies included.

**Table 6:** Safety, tolerability, adherence, and withdrawals HIIT: high-intensity interval training

Study	Intervention	Initial sample	Completed/analyzed	Withdrawals/dropouts	Completion rate	Adverse events/tolerability
Koçak et al. [18]	Balneotherapy	54	54	0 reported	100%	Not clearly reported
Kantyka et al. [21]	Aqua aerobics	21	21	0 reported	100%	Not clearly reported
Vasanth Kumar et al. [25]	Steam bath/sauna bath	60	60	0 reported	100%	No serious adverse effects reported
Tripathi et al. [27]	Steam bath/sauna bath	100	100	0 reported	100%	Not clearly reported
Sobczak et al. [19]	Aqua aerobics	30	30	0 reported	100%	Not clearly reported
LeBaron et al. [20]	Hydrogen-rich water	60	60	0 reported	100%	Well tolerated
Ben Cheikh et al. [22]	Water-based aerobic training	27	27	0 reported	100%	Not clearly reported
Liao et al. [23]	Aquatic HIIT/land HIIT	37	28	9 reported	75.7%	No detailed adverse-event data reported
Penaforte et al. [24]	Continuous/intermittent water exercise	36	27	9 reported	75.0%	Dropout reasons not recorded
Choi et al. [26]	Dry sauna	38	38	0 reported	100%	No adverse events reported

Certainty of Evidence

Randomized controlled studies with objective anthropometric and biochemical endpoints showed the highest certainty of evidence, and uncontrolled pre-post studies showed the lowest. Body weight, BMI, waist-related indices, and body fat were rated as low to moderate for evidence due to multiple studies showing a consistent directional improvement, albeit with small sample sizes and varying interventions. The evidence for lipids was weak due to the inconsistent results of the interventions by type and by unit of measurement. The reporting of adverse events was incomplete, and adherence was inconsistently documented, which resulted in low levels of evidence for tolerability and adherence. The certainty of evidence for each major outcome domain is presented in Table 7, obtained based on the consistencies, limitations, and strength of the evidence found.

**Table 7:** Certainty of evidence by outcome domain BMI: body mass index; WHR: waist-to-hip ratio; HIIT: high-intensity interval training; LDL-C: low-density lipoprotein cholesterol; HDL-C: high-density lipoprotein cholesterol

Outcome domain	Evidence pattern	Main limitation	Certainty
Body weight	Improvement mainly after active aquatic exercise and water-based aerobic training	Small samples and heterogeneous intervention protocols	Low to moderate
BMI	Reduction reported in several active and thermal intervention studies	Variable duration and comparator conditions	Low
Waist/WHR outcomes	Improvement in selected aqua aerobics, aquatic HIIT, hydrogen-rich water, steam, and sauna studies	Inconsistent measurement across studies	Low
Body fat/fat mass	Greater improvement after movement-based aquatic interventions	Different body composition assessment methods	Low
Total cholesterol	Improvement mainly in hydrogen-rich water and selected exercise studies	Inconsistent statistical significance	Low
LDL-C	Reduction seen in aqua aerobics, aquatic HIIT, hydrogen-rich water, and water aerobic training	Small sample sizes	Low to moderate
HDL-C	Mixed direction of effect across studies	Inconsistent response pattern	Low
Triglycerides	Reduction in hydrogen-rich water, steam/sauna bath, and water aerobic training	Heterogeneous interventions and reporting units	Low
Adverse events/tolerability	No major safety signal in studies reporting safety	Under-reporting of adverse events	Low
Adherence/withdrawals	Completion was high in most studies	Attendance and withdrawal reasons were inconsistently described	Low

Discussion

The current systematic review suggests that hydrotherapy-based and water-based therapy may be associated with changes in selected anthropometric and lipid parameters in people with overweight or obesity. Active aquatic exercise studies generally reported improvements in body weight, BMI, waist-related measures, body fat percentage, and fat mass; however, the magnitude and consistency of these changes varied across studies. These findings should be interpreted as descriptive rather than as evidence that active aquatic exercise is superior to passive hydrothermal exposure. This pattern indicates that the physical properties of water may improve exercise tolerance and allow individuals with excess body weight to participate in structured exercise with less joint aggravation [28]. The mechanisms underlying these benefits are likely multifactorial. Buoyancy reduces apparent body weight and decreases weight-bearing stress on the lower limbs, which may improve comfort, balance, and movement confidence in individuals with obesity [23]. Hydrostatic pressure may support venous return and cardiovascular efficiency during exercise, whereas water resistance provides continuous multidirectional loading that promotes muscle activation and energy expenditure without high-impact mechanical stress [18]. Thermal properties of water may also influence circulation, perceived exertion, and relaxation, further supporting exercise participation. Water resistance acts as a constant low-impact training stimulus and may help activate muscles and increase energy expenditure during aerobic and functional movement [29]. Aquatic HIIT and water-based aerobic training were associated with improvements in several body composition measures within the respective studies [21]. Continuous water exercise showed improvements in a greater number of anthropometric measures than intermittent water exercise within the study that directly compared these approaches; this finding should not be generalized beyond that individual comparison.

The anthropometric pattern of passive hydrothermal interventions was less uniform. Studies of steam bath and sauna bath showed immediate reductions in BMI, waist circumference, hip circumference, and triglycerides, whereas dry sauna and balneotherapy had limited effects on body composition [18,30]. These changes may reflect acute fluid shifts, sweating, peripheral vasodilation, autonomic modulation, and short-term metabolic responses rather than sustained reduction in adiposity. This mechanism helps explain why passive hydrothermal approaches may show short-term reductions in weight-related measures without producing consistent long-term changes in fat mass or BMI [29]. Balneotherapy and dry sauna appeared to be more closely associated with quality-of-life improvement than with major body composition or lipid changes. Accordingly, passive hydrothermal interventions may have supportive roles related to comfort or well-being, while the available evidence is insufficient to establish an independent effect on sustained weight or fat reduction.

Lipid profile findings were heterogeneous. In the individual studies reporting lipid outcomes, hydrogen-rich water was associated with reductions in total cholesterol, triglycerides, VLDL-C, glucose-related markers, and inflammatory parameters. These findings arose from a limited evidence base and should not be interpreted as establishing a causal metabolic effect. This intervention differs from exercise-based hydrotherapy, as its proposed effects are related to antioxidant activity, oxidative stress modulation, anti-inflammatory pathways, and metabolic signaling rather than mechanical exercise effects [31]. In obesity, oxidative stress and chronic low-grade inflammation are closely linked with insulin resistance, dyslipidemia, and cardiometabolic risk; therefore, the reported lipid and inflammatory changes with hydrogen-rich water may be biologically plausible through redox-sensitive metabolic regulation [8]. Water-based aerobic training was associated with changes in triglycerides, LDL-C, and HDL-C, while aquatic HIIT mainly reduced LDL-C, with smaller effects on total cholesterol, HDL-C, and triglycerides. These findings indicate that lipid response may depend on intervention intensity, duration, baseline metabolic profile, and the specific lipid parameter assessed. Differences in baseline characteristics and concomitant management may also have influenced these outcomes.

The available findings suggest that structured aquatic exercise may represent a feasible alternative exercise modality for selected individuals with overweight or obesity, rather than demonstrating a specific curative effect of hydrotherapy. Aerobic and interval training programs are associated with reductions in adiposity, waist circumference, insulin resistance, and atherogenic lipid fractions in people with overweight or obesity [32]. Aquatic exercise may produce comparable benefits in selected individuals by allowing sustained activity with reduced mechanical loading. This feature is clinically relevant for individuals who have musculoskeletal pain, poor exercise tolerance, balance limitations, or low confidence with land-based exercise. The adolescent aquatic HIIT study showed benefits for body mass, BMI, fat percentage, waist circumference, WHR, and LDL-C, comparable to land-based HIIT. Within that study, these findings support aquatic HIIT as an alternative mode of exercise rather than evidence of superiority over land-based training [33]. From a mechanistic perspective, the combination of reduced joint loading and preserved training intensity may allow aquatic HIIT to provide a practical means of achieving exercise-related cardiometabolic benefits in populations with excess body weight.

Safety and feasibility findings also support clinical relevance. Most studies reported high completion rates, and studies that specifically assessed adverse events did not identify major safety concerns. Hydrogen-rich water, dry sauna, steam bath, sauna bath, and supervised aquatic exercise were generally well tolerated [34,35]. These findings are important because people with obesity may have reduced exercise confidence, joint pain, impaired mobility, or limited tolerance for high-impact activity. Hydrotherapy interventions may provide a less physically demanding environment that supports participation, particularly when conventional land-based exercise is difficult. The overall evidence therefore supports consideration of active aquatic exercise as a potentially feasible complementary approach, while evidence for hydrogen-rich water and passive thermal modalities remains more limited and heterogeneous. Future trials should evaluate mechanism-linked outcomes such as energy expenditure, inflammatory biomarkers, oxidative stress markers, insulin resistance, body composition compartments, and longer-term lipid changes to clarify whether observed improvements represent true metabolic adaptation or short-term physiological shifts. The observed anthropometric and lipid changes should not be attributed solely to hydrotherapy because age, sex, baseline BMI, metabolic status, comorbid conditions, medication use, dietary regulation, and other lifestyle measures were not consistently controlled or reported across the included studies.

Limitations and Future Recommendations

The included evidence was characterized by substantial methodological and clinical heterogeneity, warranting cautious interpretation of the findings. The interventions were diverse and included active aquatic exercise as well as passive approaches such as sauna, steam bath, balneotherapy, and hydrogen-rich water, which limited direct comparison across studies. The included studies were therefore not considered to represent a single homogeneous therapeutic exposure. Differences in baseline clinical and metabolic characteristics, concomitant pharmacological treatment, dietary regulation, intervention intensity, treatment frequency, treatment duration, and comparator conditions could have modified the observed anthropometric and lipid responses. Accordingly, comparisons were restricted to descriptive patterns within broadly similar intervention categories rather than being used to infer comparative efficacy across fundamentally different hydrotherapy modalities. Sample sizes were typically small, and some studies used short intervention periods or pre-post designs with limited comparator groups. Age, sex, baseline BMI, severity of obesity, metabolic status, comorbid conditions, pharmacological treatment, dietary regulation, and other lifestyle interventions were not consistently controlled or reported across studies. These factors represent important potential confounders and limit the ability to determine whether observed anthropometric or lipid changes were independently attributable to hydrotherapy. Reporting of outcomes was inconsistent, particularly for lipid parameters, adherence, adverse events, and reasons for withdrawal. Variation in intervention intensity, session frequency, treatment cycle, duration, comparator conditions, and follow-up further limited direct comparison and causal interpretation.

Randomized controlled trials with adequate sample sizes, standardized intervention protocols, clearly defined comparator groups, and longer follow-up are recommended to improve the generalizability of future findings. Future trials should standardize or prospectively document medications, dietary intake, physical activity outside the intervention, and other concurrent lifestyle measures. Stratification or adjusted analyses according to age, sex, baseline BMI, metabolic status, and relevant comorbidities would further help distinguish the contribution of hydrotherapy from potential confounding factors. Body weight, BMI, waist circumference, WHR, body fat percentage, triglycerides, total cholesterol, LDL-C, and HDL-C should be reported consistently. Structured adverse-event monitoring, adherence reporting, and prespecified subgroup or adjusted analyses should also be incorporated into future trials. These measures would improve comparability across studies and strengthen the evidence base for hydrotherapy-based interventions in overweight and obesity.

## Conclusions

Hydrotherapy-based and water-based interventions were associated with changes in selected anthropometric and lipid outcomes in individuals with overweight or obesity, although the magnitude and consistency of these findings varied according to intervention type. Active aquatic interventions, including aqua aerobics, water-based aerobic training, aquatic HIIT, and continuous water exercise, generally showed improvements in body weight, BMI, waist-related measures, body fat percentage, and fat mass; however, these findings should not be interpreted as evidence of superiority across interventions because of heterogeneity among the included studies. Hydrogen-rich water was associated with reductions in BMI, WHR, total cholesterol, triglycerides, and selected cardiometabolic markers in the limited available evidence. Passive hydrothermal methods such as balneotherapy, steam bath, sauna bath, and dry sauna exhibited more inconsistent results, with mainly short-term anthropometric changes or improvements in quality of life rather than consistent effects on body composition or lipid parameters. The safety findings were generally reassuring, although reporting of tolerability, adherence, adverse events, and withdrawals was inconsistent. Hydrotherapy-based interventions may have a complementary role in obesity management, particularly when land-based exercise is difficult; however, the available evidence does not establish an independent therapeutic or curative effect because of clinical heterogeneity, potential confounding, and inconsistent control of concomitant dietary, pharmacological, and lifestyle factors.
